# Effect of printing orientation on mechanical properties of 3D-printed orthodontic aligners

**DOI:** 10.1007/s00056-023-00511-0

**Published:** 2024-01-26

**Authors:** Lukas Camenisch, Georgios Polychronis, Nearchos Panayi, Olga Makou, Spyridon N. Papageorgiou, Spiros Zinelis, Theodore Eliades

**Affiliations:** 1 Clinic of Orthodontics and Pediatric Dentistry, Center for Dental Medicine, University of Zurich, Plattenstr. 11, 8032 Zurich, Switzerland; 2 Department of Biomaterials, School of Dentistry, National and Kapodistrian University of Athens, Athens, Greece; 3 Department of Dentistry, European University Cyprus, Nicosia, Cyprus

**Keywords:** Three-dimensional printing, Orthodontic aligner, Instrumented indentation testing, Mechanical properties, Removable orthodontic appliances, Dreidimensionaler Druck, Kieferorthopädische Aligner, Instrumentierte Indentationsprüfung, Mechanische Eigenschaften, Herausnehmbare kieferorthopädische Geräte

## Abstract

**Purpose:**

The purpose of this study was to assess differences in the fundamental mechanical properties of resin-made three-dimensional (3D) printed orthodontic aligners according to the printing orientation.

**Methods:**

Twenty resin 3D-printed dumbbell-shaped specimens and 20 orthodontic aligners were fabricated and postcured in nitrogen. Half of the specimens and aligners were built in horizontal (H), the other half in vertical (V) directions. The dumbbell-shaped specimens were loaded in a tensile testing machine, while parts of the aligners were embedded in acrylic resin, ground, polished, and then underwent instrumented indentation testing (IIT). Mechanical properties that were assessed included the yield strength (YS), breaking strength (BS), plastic strain (ε), Martens hardness (HM), indentation modulus (E_IT_), elastic index (η_IT_), and indentation relaxation (R_IT_). Data were analyzed statistically with independent t‑tests or Mann–Whitney tests at α = 5%.

**Results:**

No significant differences were found between specimens or aligners printed either in a horizontal or a vertical direction (*P* > 0.05 in all instances). Overall, the 3D-printed aligners showed acceptable mechanical propertied in terms of YS (mean 19.2 MPa; standard deviation [SD] 1.7 MPa), BS (mean 19.6 MPa; SD 1.2 MPa), ε (mean 77%; SD 11%), HM (median 89.0 N/mm^2^; interquartile range [IQR] 84.5–90.0 NN/m^2^), E_IT_ (median 2670.5 MPa; IQR 2645.0–2726.0 MPa), η_IT_ (median 27.5%; IQR 25.9–28.1%), and R_IT_ (mean 65.1%; SD 3.5%).

**Conclusion:**

Printing direction seemed to have no effect on the mechanical properties of 3D-printed resin aligners, which are promising for orthodontic use.

## Abstract (de)

**Zweck:**

Ziel dieser Studie war es, die Unterschiede in den grundlegenden mechanischen Eigenschaften von aus Kunststoff hergestellten dreidimensionalen (3-D) gedruckten kieferorthopädischen Alignern in Abhängigkeit von der Druckausrichtung zu untersuchen.

**Methoden:**

Zwanzig aus Kunststoff 3‑D-gedruckte hantelförmige Prüfkörper und 20 kieferorthopädische Aligner wurden hergestellt und in Stickstoff nachgehärtet. Die Hälfte der Prüfkörper und Aligner wurde in horizontaler, die andere Hälfte in vertikaler Richtung hergestellt. Die hantelförmigen Prüfkörper wurden in einer Zugprüfmaschine belastet, während Teile der Aligner in Acrylharz eingebettet, geschliffen und poliert und anschließend einer instrumentierten Eindruckprüfung (IIT) unterzogen wurden. Zu den bewerteten mechanischen Eigenschaften gehörten die Streckgrenze (YS), die Bruchfestigkeit (BS), die plastische Dehnung (ε), die Martens-Härte (HM), der Eindringmodul (EIT), der Elastizitätsindex (ηIT) und die Eindringrelaxation (RIT). Die Daten wurden mit unabhängigen t‑Tests oder Mann-Whitney-Tests bei α = 5% statistisch ausgewertet.

**Ergebnisse:**

Es wurden keine signifikanten Unterschiede zwischen den in horizontaler oder vertikaler Richtung gedruckten Proben oder Alignern festgestellt (*p* > 0,05 in allen Fällen). Insgesamt zeigten die 3‑D-gedruckten Aligner akzeptable mechanische Eigenschaften in Bezug auf YS (Mittelwert 19,2 MPa; Standardabweichung [SD] 1,7 MPa), BS (Mittelwert 19,6 MPa; SD 1,2 MPa), ε (Mittelwert 77%; SD 11%), HM (Median 89,0 N/mm^2^; Interquartilsbereich [IQR] 84,5-90,0 NN/m^2^), EIT (Median 2670,5 MPa; IQR 2645,0-2726,0 MPa), ηIT (Median 27,5%; IQR 25,9-28,1%) und RIT (Mittelwert 65,1%; SD 3,5%).

**Schlussfolgerung:**

Die Druckrichtung schien keinen Einfluss auf die mechanischen Eigenschaften von 3‑D-gedruckten Kunststoff-Alignern zu haben, was für die kieferorthopädische Anwendung vielversprechend ist.

## Introduction

Since the introduction of three-dimensional (3D) printing technologies in orthodontics, direct fabrication of orthodontic aligners has emerged as a promising alternative and has gathered the interest of clinicians and researchers alike. This highly efficient do-it-yourself method offers considerable advantages, such as same day availability of appliances within the orthodontic office at a lower cost by circumventing the dental laboratory, while giving direct control to the orthodontist, better geometrical accuracy, and improved efficacy [11, 29]. In contrast to the indirect technique, where aligners are thermoformed on models, directly 3D-printed aligners are formed layer by layer based on digital 3D models. The majority of available 3D printers utilize a light source capable of polymerizing resins in vertical, diagonal, or horizontal directions [19]. The process is quickest per aligner for the horizontal orientation, due to the smaller number of layers added by the 3D printer and the reduced lines to the aligner’s anterior teeth labial surfaces [18]. On the other hand, vertical printing favors a larger appliance volume production per single print job. Printing supports are also distributed differently according to the chosen orientation of the aligner during printing. It seems, however, that independent of the type of 3D printer used, 3D printing procedures using either horizontal or vertical orientation seem to be accurate and satisfy patient demands.

However, additive manufacturing techniques have the inherent disadvantage of potentially incorporating stresses between the material layers, which is attributable to shrinkage of the material after layering [4, 14, 31]. The direction of aligner printing may therefore influence the magnitude and orientation of these residual stresses. This phenomenon is a well-known side effect found in the 3D printing of metallic components [27] and is, at least to some degree, addressed by postthermal treatment [4]. Unfortunately, this treatment can be used on neither thermoformed aligners nor on resin-printed ones. The introduction of such internal stresses may lead to alterations of their major mechanical properties including hardness, elastic relaxation, and deformation. These changes are not to be taken lightly, since the clinical efficiency of the fabricated orthodontic appliances is largely dependent on them. In particular, the modulus of elasticity [9, 32, 33] and elastic relaxation [6, 10, 17] are crucial for the aligners to be able to exert light and continuous forces on the teeth, while hardness is related to the aligner’s resistance to abrasion [33]. Furthermore, the printing direction may have an adverse effect on surface roughness, which promotes appliance plaque accumulation and discoloration [13, 15] and might ultimately influence the, sometimes questionable [9, 21], clinical performance of aligners.

There is a lack of scientific evidence in the published literature regarding the mechanical properties of orthodontic aligners printed with different orientations. Thus, the current choice for 3D printing of such aligners is based mostly on efficiency aspects in terms of speed and mass production, and the actual efficacy of the end product in use is disregarded. Therefore, the aim of this study was to investigate the effect of printing orientation on the material mechanical properties of 3D manufactured orthodontic aligners. The null hypothesis was that there is no difference in the mechanical properties of orthodontic aligners directly printed with a 3D process in either a horizontal or a vertical orientation.

## Materials and methods

### Sample preparations

**Fig. 1:**
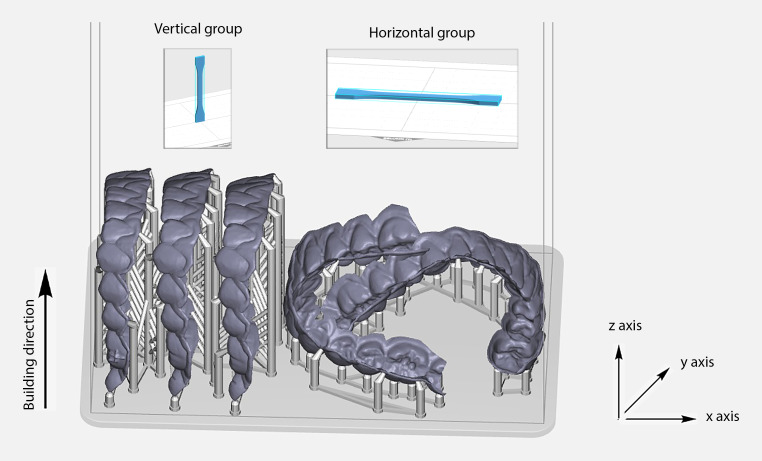
An illustration showing the orientation of the vertical and horizontal groups over an x‑y table and building direction (z-axis) Eine Illustration, die die Ausrichtung der vertikalen und horizontalen Gruppen über einen x‑y-Tisch und die Baurichtung (z-Achse) zeigt

Twenty dumbbell-shaped specimens and 20 full-arch orthodontic aligners were fabricated using the SprintRay Pro 95 (SprintRay, Los Angeles, CA, USA) 3D printer utilizing direct light processing technology. Ten of the specimens and 10 of the aligners were printed in horizontal direction, starting from the cervical towards the incisal regions (group H). The other 10 specimens/aligners were printed in vertical direction, starting from the distal towards the mesial regions (group V), as illustrated in Fig. 1. The printer tank was filled with Tera Harz TC-85DAC resin (Tera Harz, Graphy, Seoul, Korea) indicated for the manufacturing of clear aligners. The aligners were printed in successive layers of 100 μm nominal size in 70 min, employing a 405 nm blue–violet light. A centrifugation machine was used to remove excess resin for 4 min and then both specimens and aligners were postcured for 14 min from both sides (cervical and incisal side for aligners) in a postcuring unit. This nitrogen generator Tera Harz Cure THC2 (Graphy, Seoul, Republic of Korea) is able to produce a continuous nitrogen from dry compressed air, thereby resulting in an oxygen-free atmosphere in order to minimize oxygen inhibition phenomena and to increase polymerization. Finally, printed supporting columns were removed and the specimens/aligners were polished at the points of supports using a handpiece with polishing brushes.

### Tensile testing

The width and thickness of each dumbbell shaped specimen was measured by a digital micrometer (Mitutoyo, Kanagawa, Japan) and then was loaded up to its fracture with a universal tensile testing machine at a crosshead speed of 1 mm/min (Tensometer 10, Monsanto, Swindon, UK). The yield strength (YS), the breaking strength (BS), and the plastic strain (ε) were calculated from the stress–strain curve.

### Instrumented indentation testing

The mandibular first molars of the fabricated aligners for groups H and V were cut off from the appliances and the specimens were embedded in acrylic resin (Verso Cit‑2, Struers, Ballerup, Denmark), with their occlusal surfaces parallel to the horizontal plane. The samples were then ground using up to 4000 grit-size SiC papers under water cooling and polished with a water-based diamond suspension (NapR1 DiaPro, Struers) of 1 μm particle size in a grinding/polishing machine (Dap‑V, Struers, Ballerup, Denmark). Instrumented indentation testing (IIT) was used to measure the mechanical properties in both groups, including Martens hardness (HM), indentation modulus (E_IT_), elastic index (η_IT_), and indentation relaxation (R_IT_). Testing was conducted in a universal hardness testing machine (ZHU0.2/Z2.5, Zwick Roell, Ulm, Germany) with a Vickers indenter, applying two different loading regimes. The HM, E_IT_ and η_IT_ were determined automatically from force–indentation depth curves, applying a maximum load of 4.9 N for a 2-second contact time. For R_IT_, a rectangular force pulse with a constant indentation depth of 50 μm was applied for 120 s with a total monitoring time of approximately 130 s. The R_IT_ value was measured by monitoring the decrease in force between the start and the end of the constant indentation depth period. All mechanical properties were calculated according to the international standard ASTM E 2546-15 by the National Institute of Standards and Technology [30] using a Poisson’s ratio of 0.357 [23].

### Statistical analysis

The distribution of continuous variables was checked through visual plot inspection and formally with the Shapiro–Wilk test. Descriptive statistics included mean with standard deviation (SD) for normally distributed and median with interquartile ranges (IQR) for skewed data. Differences between horizontally and vertically printed specimens were assessed with independent-samples t‑test for normally distributed (after checking for variance-equality with the Brown–Forsythe test) or Mann–Whitney test for skewed data. All analyses were done with a two-sided alpha (α) at 5%, in Stata SE 14.2 (StataCorp, College Station, TX, USA), and with an openly provided dataset [2].

## Results

**Fig. 2:**
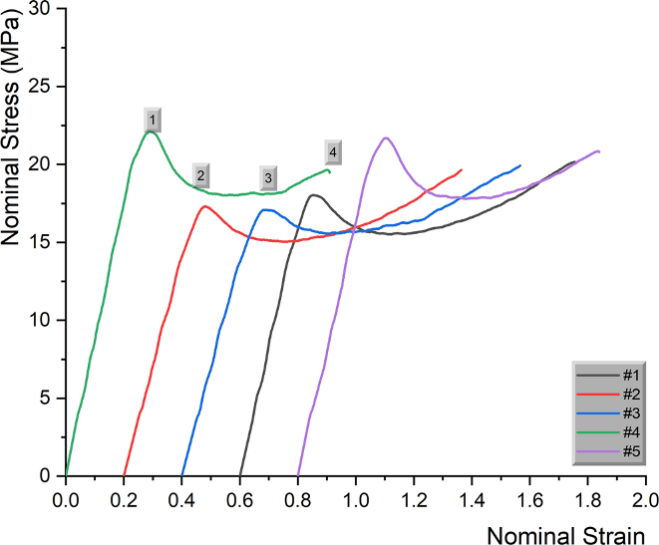
Representative stress strain curves from both groups. Five curves (specimens #1 to #5) from the vertical (V) group are presented. Numbers over *green curve* (#4) indicate characteristic points of stress strain curves: (*1*) Yield point and initiation of local decrease in cross-sectional area (neck); (*2*) After necking, the nominal stress falls at a constant value as the neck extends along the full gauge length of dumbbell and the polymer chains tend to align themselves parallel to the direction of tensile stress; (*3*) Neck has been spread to the full gauge length and thanks to orientation of molecular chains is stronger and stiffer and the stress start, rising again up to final fracture (*4*). The curves are presented with a 0.2 offset for the sake of clarity Repräsentative Spannung-Dehnung-Kurven aus beiden Gruppen. Es werden 5 Kurven (Proben #1 bis #5) aus der vertikalen (V) Gruppe dargestellt. Die Zahlen über der *grünen Kurve* (#4) geben charakteristische Punkte der Spannungs-Dehnungs-Kurven an: (*1*) Streckgrenze und Beginn der lokalen Abnahme der Querschnittsfläche („neck“, Hals); (*2*) Nach der Verengung („necking“) fällt die Nennspannung auf einen konstanten Wert, da sich die Verengung über die gesamte Messlänge der Hantel erstreckt und die Polymerketten dazu neigen, sich parallel zur Richtung der Zugspannung auszurichten; (*3*) der Hals hat sich über die gesamte Messlänge ausgebreitet und ist dank der Ausrichtung der Molekülketten stärker und stabiler, die Spannung beginnt wieder anzusteigen, bis zum endgültigen Bruch am Punkt (*4*). Die Kurven sind der Übersichtlichkeit halber um 0,2 versetzt dargestellt

Representative stress–strain curves from both groups are presented in Fig. 2. In all instances, stresses increased steadily up to the yield point (point 1) and a local decrease in the specimen’s cross-sectional area started. After necking, the nominal stress started falling at a constant value as the neck extended along the full gauge length of the dumbbell and the polymer chains tended to align themselves parallel to the direction of tensile stress (point 2). Afterwards, the neck started to spread to the full gauge length (point 3) and, thanks to orientation of molecular chains, was stronger/stiffer and the stresses started to rise again up to final fracture (point 4). Several mechanical properties were measured from the tensile testing of the included dumbbells, including yield strength (mean 19.2 MPa; SD 1.7 MPa), breaking strength (mean 19.6 MPa; SD 1.2 MPa), and plastic strain (mean 0.77%; SD 0.11%), with no statistically significant differences between the horizontal and vertical group (*P* > 0.05 in all instances; Table 1).

**Table 1:** Mechanical properties of specimens printed horizontally or vertically Mechanische Eigenschaften von horizontal oder vertikal gedruckten Prüfkörpern

Group	YS (MPa)	BS (MPa)	ε (%)	HM (N/mm^2^)	E_IT_ (MPa)	η_ΙΤ_ (%)	R_IT_ (%)
Metric	Mean (SD)	Mean (SD)	Mean (SD)	Median [IQR]	Median [IQR]	Median [IQR]	Mean (SD)
Horizontal	19.7 (1.8)	19.6 (1.0)	0.76 (0.13)	86.5 [83.0, 90.0]	2682.5 [2655.0, 2726.0]	26.7 [25.7, 28.1]	66.2 (3.5)
Vertical	18.8 (1.6)	19.6 (1.4)	0.78 (0.09)	89.0 [88.0, 91.0]	2656.0 [2617.0, 2726.0]	27.8 [27.2, 28.0]	63.9 (3.2)
*P*	0.30^a^	0.99^a^	0.66^a^	0.32^b^	0.21^b^	0.60^b^	0.14^a^

*BS* breaking strength, *E*_*IT*_ indentation modulus, *HM* Martens hardness, *IQR* interquartile range, *R*_*IT*_ indentation relaxation, *SD* standard deviation, *YS* yield strength, *ε* plastic strain, *η*_*IT*_ elastic index

^a^From t‑test for independent samples

^b^From Mann–Whitney test

**Fig. 3:**
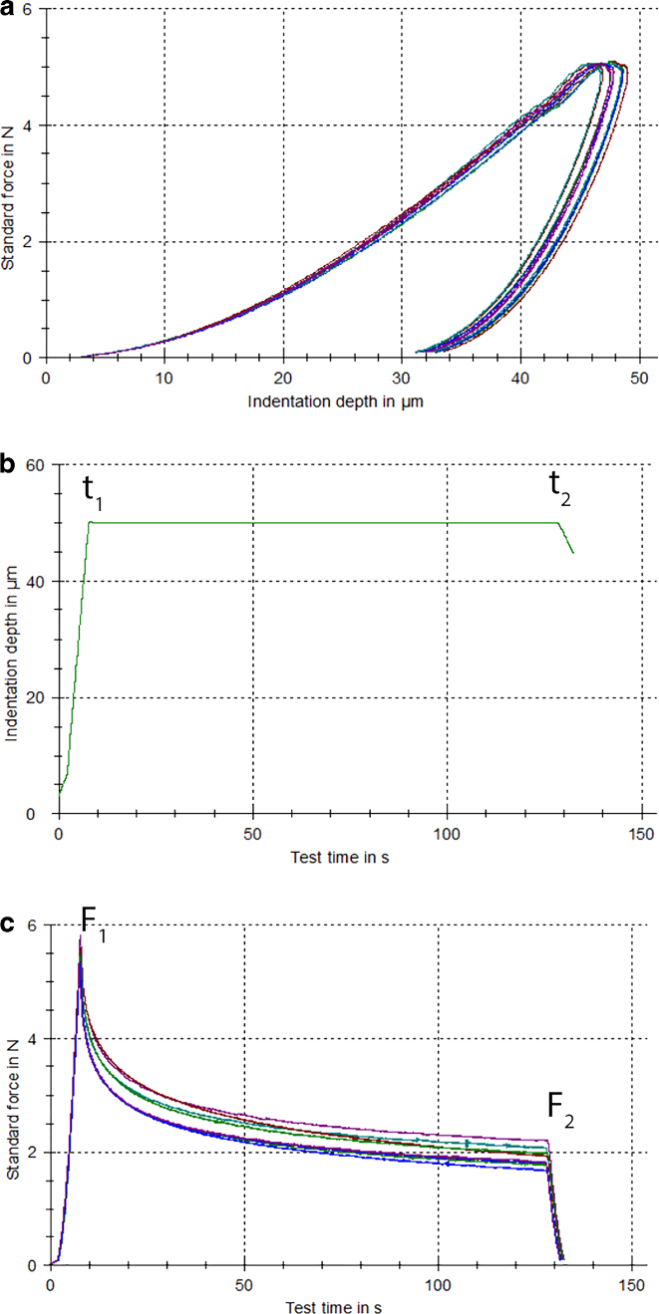
**a** Representative force indentation depth curves from both groups tested; **b** rectangular pulse of standard indentation depth of 50 μm for 120 s applied for the measurement of relaxation index with a total monitoring time of approximately 130 s; **c** representative curves of force decay over time of material tested during application of rectangular pulse **a** Repräsentative Kraft-Eindringtiefe-Kurven von beiden getesteten Gruppen; **b** rechteckiger Impuls mit einer Standardeindringtiefe von 50 μm für 120 s zur Messung des Relaxationsindexes mit einer Gesamtüberwachungszeit von etwa 130 s; **c** repräsentative Kurven des Kraftabfalls über die Zeit des geprüften Materials während der Anwendung des rechteckigen Impulses

Force-indentation depth curves for both groups are illustrated in Fig. 3a. Several mechanical properties were measured from IIT of the included aligners, including Martens hardness (median 89.0 N/mm^2^; IQR 84.5–90.0 N/mm^2^), indentation modulus (median 2670.5 MPa; IQR 2645.0–2726.0 MPa), elastic index (median 27.5%; IQR 25.9–28.1%), and indentation relaxation (mean 65.1%; SD 3.5%; Table 1), with no statistically significant differences between the horizontal and vertical group (*P* > 0.05 in all instances; Table 1). Finally, Fig. 3b presents the rectangular pulse applied with a period of standard indentation depth, while Fig. 3c depicts the degradation of the applied force over the experimental time.

## Discussion

The results of this in vitro study do not provide adequate justification to reject the null hypothesis of no difference between horizontal and vertical printing orientation for orthodontic aligners. This study employed both tensile testing and IIT to provide a full characterization of the wide spectrum of possible effect of printing orientation on total volume (tensile testing) and micro scale (IIT) of the material tested. Contrary to tensile testing, where bulky specimens are required, IIT can provide information on the mechanical properties of small and irregular specimens such as aligners [33].

No statistically significant differences were identified for YS, BS, and ε (Table 1) between the horizontal and the vertical group, implying that the printing orientation has no effect on tensile properties of the printed aligners. All stress–strain curves (Fig. 2) presented the characteristic shape of a polymeric tensile curve with four characteristic points: (*1*) yield point and initiation of local decrease in cross-sectional area (neck); (*2*) after necking, the nominal stress falls at a constant value as the neck extends along the full gauge length of dumbbell. This process is known as cold drawing and the polymer chains tend to align themselves parallel to the direction of tensile stress, resulting in a strain-hardening mechanism; (*3*) neck has been spread to the full gauge length, becomes stronger/stiffer as the molecular chains are fully aligned, and stresses start to develop; and (*4*) rise again up to final fracture (4) [26].

The average modulus of elasticity for both aligner groups (median of 2670.5 MPa) was similar to previous studies, in which resin-made 3D-printed aligners were fabricated by different printers and under different conditions [3, 33]. The same was true when the results are compared to the polyurethane-made Invisalign® (Align Technology, Santa Clara, CA, USA) aligners [8, 20]. Conversely, the modulus of elasticity measured in the present study was higher than that reported from thermoformed aligners made of polyethylene terephthalate glycol (PET‑G; 1530 to 2370 MPa [1, 5, 28]). The increased resistance to elastic deformation of resins is associated with a higher magnitude of forces transmitted to the teeth upon aligner use. From a clinical point of view this means that 3D-printed aligners can be thinner, and therefore more patient-tolerated than their thermoformed counterparts, while being equally effective under the same strain [1, 12]. Concerning HM, the 3D-printed resin aligners displayed similar hardness (median of 89 N/mm^2^) to previously reported PET‑G aligners (92–101 N/mm^2^) [1] and resin aligners (94–108 N/mm^2^) [3, 33]. However, the material of Invisalign® aligners seems to be harder (118–122 N/mm^2^) [1, 8, 20] and could consequently be more resistant to intraoral wear. On the other hand, the small mean values of the 3D-printed resins’ elastic index (27.5%) emphasize their ductile nature [3], which is in accordance with the tensile results where a 0.75 (75%) plastic strain was found after fracture (Table 1). In contrast to the values of Invisalign® aligners (40%) [8, 20] and PET‑G (35%) aligners [1], 3D-printed aligners are less brittle and therefore less prone to fracture upon removal. In addition, 3D-printed resins showed a lower resistance to relaxation, given the high values of the relaxation index (65.1%) compared to the low values of Invisalign® (~ 4%) [20]. This is associated with a more dramatic force decrease under constant deformation for 3D printed than for Invisalign® aligners, agreeing with previous data [3], and which may be initially seen as problematic from an orthodontic treatment perspective. However, the high magnitude of initial forces applied to the teeth, due to the resin’s increased modulus of elasticity, needs to decrease rapidly to the ideal light and constant orthodontic forces, which are essential for efficient tooth movement [32]. Hence, an increased relaxation index might ultimately prove to be beneficial in this respect—but this requires further testing under in vivo conditions.

The results of both tensile and IIT testing agreed that printing orientation had no effect on the mechanical properties of 3D-printed aligners. This is in contrast with older studies reporting that printing orientation using stereolithography (SLA) had a significant effect on the mechanical properties of 3D-printed resin dumbbell specimens [16, 24, 25] and therefore appliances printed with SLA cannot be considered isotropic. On the other hand, data from a more recent study indicated that SLA printing with different orientations did not significantly influence the mechanical properties of printed resin dumbbell specimens [27]. It seems that the postcure process and the specific conditions during the aligner’s manufacturing may alleviate any residual stresses in the manufactured aligner and thereby affect the mechanical properties and clinical performance of the aligner. Unlike metallic 3D-printed components, which are characterized by high anisotropy in all axes [7] and where the printing orientation dictates the spatial orientation of the printed material, the resin material is not fully set anisotropic after printing. Thus, enough time is given for the polymeric chains to spatially rearrange, counteracting orientation differences, while simultaneously polymerization proceeds in multiple directions and more importantly in depth without the presence of oxygen inhibition phenomena—the latter augmented by the highly favorable nitrogen gas environment [22]. Therefore, it seems that any spatial orientation differences of the printed material are negated by the postcuring process, which was similar for the two groups. Nevertheless, future research that includes structural and chemical characterization of 3D-printed components under various conditions and configurations (including different postcuring processes) may shed further light on this.

In conclusion, the 3D-printed resin aligners tested showed similar mechanical properties to the material of Invisalign® aligners and thermoplastic materials and, thus, should be considered as a promising candidate for orthodontic tooth movements. However, there is still much room for additional investigations on biocompatibility issues and aging effects, which will surely pave the road for the optimization of these recently introduced materials.

## Conclusion

The results of the present in vitro study indicate that printing orientation (horizontal or vertical) has no significant effect on the mechanical properties of three-dimensional (3D)-printed aligners from resin. The clinical implications of these results are that clinicians might consider 3D-printed aligners to be an isotropic material and, thus, a similar mechanical reaction might be anticipated intraorally under multidimensional activation.
